# Urgency of a political agenda for the reclassification of hazard pay

**DOI:** 10.1590/0034-7167.20267906

**Published:** 2026-08-17

**Authors:** Maria Helena Palucci Marziale, Jacinta de Fatima Sena da Silva

**Affiliations:** I Full Professor, Escola de Enfermagem de Ribeirão Preto, Universidade de São Paulo. Director of Publications of the Brazilian Nursing Association. Ribeirão Preto, São Paulo, Brazil; II President of the Brazilian Nursing Association. Brasília, Federal District, Brazil

The Brazilian nursing workforce, a fundamental pillar of healthcare delivery, has historically faced a paradox between the essential nature of its work for society and the regulatory framework governing its working conditions. In this editorial, we discuss the hazard pay allowance established in Brazil by Regulatory Standard 15 (RS-15)^(1)^ of the Ministry of Labor, which imposes a limitation by restricting the maximum degree of hazardous exposure (40%) exclusively to isolation environments and to the safety regulations established by RS-32^(2)^. These regulations are contrasted with evidence-based arguments from the literature derived from epidemiological analyses of exposure resulting from work processes and conditions involving biological risk^(3-6)^, as well as with a bioethical reflection on hazard pay under the principle of human dignity^(7)^.

The statistics demonstrate that although prevention and control measures minimize health problems arising from exposure to biological hazards-the primary source of hazardous exposure and illness among healthcare workers, particularly nursing professionals-the legally established measures for controlling such exposure, including the biosafety guidelines of RS-32^(2)^, are capable of mitigating adverse outcomes but remain insufficient to eliminate the risk of infections resulting from work processes and conditions involving direct contact with contaminated biological fluids. This reality explains the continued occurrence of work absences despite current preventive protocols.

RS-15^(1)^ links the recognition of biological hazardous exposure at the maximum level to the existence of physical isolation within the work environment, that is, an architectural criterion that disregards the actual epidemiological dynamics of healthcare services, such as triage units, emergency departments, and urgent care units, which are, by definition, environments characterized by continuous and unpredictable contact with pathogenic agents^(6)^.

Exposure is not confined to isolated rooms; rather, it is ubiquitous, diffuse, and constant throughout the entire work shift, as nursing professionals perform their duties under a permanent causal relationship with biological agents, in which exposure is the rule rather than a geographical exception. Therefore, the “isolation” criterion established by RS-15 was conceived within a hospital context that does not reflect contemporary epidemiological complexity^(6)^.

The premise that the risk of infectious disease transmission is confined to isolation areas is scientifically obsolete. Recent studies^(3-4)^ demonstrate that triage units and emergency departments present pathogenic burdens equivalent to or even greater than those found in isolation areas due to the constant flow of undiagnosed patients and the coexistence of multiple pathogens.

Manzi^(6)^, a judge of the Regional Labor Court, denounces the invisibility of risk in hospital triage sectors, where the absence of a prior diagnosis does not eliminate biological hazards but instead makes them more insidious. He argues that the science of care requires hazardous exposure to be assessed according to biological burden and the nature of exposure rather than architectural demarcations.

Additionally, Ezaias, Cardoso, and Marziale^(7)^ support the need to grant the maximum level of hazard pay to nursing workers under the principle of human dignity, arguing that worker health protection must be comprehensive and that differentiating hazard pay based on architectural criteria violates ethics and distributive justice. According to these authors, bioethical reflection supports the understanding that hazard pay, under the principle of human dignity, should not be viewed as a price paid for exposure to danger, but rather as a mechanism that reveals shortcomings in collective protection measures. Human dignity is the axiological core that should guide the RSs, preventing healthcare professionals’ health from being subjected to a market-oriented logic in which life is assigned a monetary value based on percentages of the minimum wage.

Therefore, the reclassification of hazardous exposure is an imperative of social justice and health safety. Protecting nursing workers is inseparable from the quality of healthcare provided to society. It is time to align legislation with science and transform evidence into a political agenda that ensures dignity and recognition for those who sustain Brazilian public health.

The convergence of scientific evidence, constitutional foundations, favorable case law, and institutional mobilization forms a robust and multidimensional argument, making the reclassification of hazardous exposure not only legitimate but also legally sustainable and politically urgent. This reclassification represents not merely a financial achievement, but also the formal recognition of the real risks faced daily by nursing professionals, constituting a structural step toward greater professional recognition and appreciation.

The time for the technical review of Annex 14 of RS-15 by the Ministry of Labor is now. Inaction by the profession represents the perpetuation of a regulatory injustice with a direct impact on millions of nursing professionals. Let us work together to lead the production of epidemiological indicators capable of supporting the technical review of Annex 14 of RS-15 by the Ministry of Labor, transforming data into institutional action and a tool for collective mobilization.
