# Supplementary material for: Urgency of a political agenda for the reclassification of hazard pay
Source: Rev Bras Enferm. 2026 Aug 17;79:e7906. doi: 10.1590/0034-7167.20267906 (PMC13481038; doi:10.1590/0034-7167.20267906)
Supplement: Translation

# Translation: Urgência de uma agenda política para reclassificação do adicional de insalubridade

**DOI:** 10.1590/0034-7167.20267906pt

**Authors:** Maria Helena Palucci Marziale, Jacinta de Fatima Sena da Silva

**Affiliations:** I Professora Titular da Escola de Enfermagem de Ribeirão Preto da Universidade de São Paulo. Diretora de Publicações da Associação Brasileira de Enfermagem. Ribeirão Preto, São Paulo, Brasil; II Presidenta da Associação Brasileira de Enfermagem. Brasília, Distrito Federal, Brasil

A enfermagem brasileira, pilar fundamental da atenção à saúde, enfrenta historicamente um paradoxo entre a essencialidade de seu trabalho para a sociedade e a moldura normativa que regula suas condições de trabalho. Neste editorial, discutimos o adicional de insalubridade, estabelecido no Brasil pela Norma Regulamentadora nº 15 (NR-15)^(1)^ do Ministério do Trabalho, que impõe uma barreira à restrição do grau máximo de insalubridade (40%) apenas a ambientes de isolamento e às normativas de segurança da NR-32^(2)^, contrapondo-as aos argumentos baseados em evidências da literatura a partir de análise epidemiológica da exposição advinda de processos e condições trabalho ao risco biológico^(3-6)^ e na reflexão bioética do adicional de insalubridade sob a égide do princípio da dignidade da pessoa humana^(7)^.

As estatísticas demonstram que, embora as medidas de prevenção e controle minimizem os agravos decorrentes da exposição a riscos biológicos - a principal fonte de insalubridade e adoecimento de trabalhadores da saúde, especialmente os da área de enfermagem -, as medidas legalmente estabelecidas para o controle dessa exposição, como as diretrizes de biossegurança da NR-32^(2)^, conseguem mitigar os agravos, mas são insuficientes para zerar o risco de infecções advindas de processos e condições trabalho pelo contato direto com fluidos biológicos contaminados, o que valida a persistência dos afastamentos laborais, mesmo diante dos protocolos preventivos atuais.

A NR-15^(1)^ vincula o reconhecimento da insalubridade biológica no grau máximo à existência de isolamento físico do ambiente de trabalho, ou seja, um critério de natureza arquitetônica que desconsidera a dinâmica epidemiológica real dos serviços de saúde, como unidades de triagem, pronto-socorro e unidades de emergência, que são, por definição, espaços de contato contínuo e imprevisível com agentes patogênicos^(6)^.

A exposição não é confinada a salas isoladas; ela é ubíqua, difusa e constante ao longo de toda a jornada de trabalho, pois o profissional de enfermagem atua sob um nexo causal permanente com agentes biológicos, em que a exposição é a norma, não a exceção geográfica. Portanto, o critério de “isolamento” da NR-15 foi concebido em um contexto hospitalar que não reflete a complexidade epidemiológica contemporânea^(6)^.

A premissa de que o risco infectocontagioso está confinado a áreas de isolamento é cientificamente obsoleta. Estudos recentes^(3-4)^ demonstram que unidades de triagem e prontos-socorros apresentam cargas patogênicas equivalentes ou superiores às áreas de isolamento, devido ao fluxo constante de pacientes não diagnosticados e à coexistência de múltiplos patógenos.

Manzi^(6)^, desembargador do Tribunal Regional do Trabalho, denuncia a invisibilidade do risco nos setores de triagem hospitalar, onde a ausência de diagnóstico prévio não anula a periculosidade biológica, mas a torna mais insidiosa. Ele afirma que a ciência do cuidado exige que a insalubridade seja avaliada pela carga biológica e pela natureza da exposição, e não por demarcações arquitetônicas.

Complementarmente, Ezaias, Cardoso e Marziale^(7)^ fundamentam a necessidade do grau máximo do adicional de insalubridade aos trabalhadores de enfermagem sob a égide do princípio da dignidade da pessoa humana, argumentando que a proteção à saúde do trabalhador deve ser integral e que a diferenciação de adicionais baseada em critérios arquitetônicos fere a ética e a justiça retributiva. Segundo os referidos autores, a reflexão bioética fundamenta que o adicional de insalubridade, sob a égide do princípio da dignidade da pessoa humana, não deve ser visto como um preço pago pela exposição ao perigo, mas como um mecanismo que revela a falha nas medidas de proteção coletiva. A dignidade humana é o núcleo axiológico que deve orientar as NRs, impedindo que a saúde do profissional seja submetida a uma lógica mercantilista na qual a vida é precificada em percentuais sobre o salário mínimo.

Portanto, a reclassificação da insalubridade é um imperativo de justiça social e segurança sanitária. A proteção do trabalhador de enfermagem é indissociável da qualidade do cuidado de saúde prestado à sociedade. É tempo de alinhar a legislação à ciência e transformar a evidência em uma agenda política que garanta a dignidade e o reconhecimento a quem sustenta a saúde pública brasileira.

A convergência de evidências científicas, fundamentos constitucionais, jurisprudência favorável e mobilização institucional forma um argumento robusto e multidimensional, tornando a reclassificação da insalubridade não apenas legítima, mas também juridicamente sustentável e politicamente inadiável. Essa reclassificação representa não apenas uma conquista financeira, mas também o reconhecimento formal do risco real enfrentado diariamente pelos profissionais de enfermagem, constituindo um passo estrutural em direção à valorização da categoria.

O momento da revisão técnica do Anexo 14 da NR-15 junto ao Ministério do Trabalho é agora. A inação da categoria representa a perpetuação de uma injustiça normativa com impacto direto sobre milhões de profissionais de enfermagem. Vamos liderar juntos a produção de indicadores epidemiológicos que subsidiem a revisão técnica do Anexo 14 da NR-15 junto ao Ministério do Trabalho, transformando dados em ação institucional e ferramenta de apoio à mobilização coletiva.

# Translation: Urgencia de una agenda política para la reclasificación del adicional por insalubridad

**DOI:** 10.1590/0034-7167.20267906es

**Authors:** Maria Helena Palucci Marziale, Jacinta de Fatima Sena da Silva

**Affiliations:** I Profesora Titular de la Escola de Enfermagem de Ribeirão Preto de la Universidade de São Paulo. Directora de Publicaciones de la Asociación Brasileña de Enfermería. Ribeirão Preto, São Paulo, Brasil; II Presidenta de la Asociación Brasileña de Enfermería. Brasília, Distrito Federal, Brasil

La enfermería brasileña, pilar fundamental de la atención de la salud, enfrenta históricamente una paradoja entre la esencialidad de su trabajo para la sociedad y el marco normativo que regula sus condiciones laborales. En este editorial, discutimos el adicional por insalubridad establecido en Brasil por la Norma Reguladora nº 15 (NR-15)^(1)^ del Ministerio de Trabajo, que impone una limitación al restringir el grado máximo de insalubridad (40%) únicamente a ambientes de aislamiento y a las normativas de seguridad de la NR-32^(2)^, contraponiéndolas a los argumentos basados en evidencias de la literatura, derivados de análisis epidemiológicos de la exposición proveniente de procesos y condiciones de trabajo asociados al riesgo biológico^(3-6)^, así como a la reflexión bioética sobre el adicional por insalubridad bajo el principio de la dignidad de la persona humana^(7)^.

Las estadísticas demuestran que, aunque las medidas de prevención y control minimizan los daños derivados de la exposición a riesgos biológicos -principal fuente de insalubridad y enfermedad entre los trabajadores de la salud, especialmente los profesionales de enfermería-, las medidas legalmente establecidas para controlar dicha exposición, como las directrices de bioseguridad de la NR-32^(2)^, logran mitigar los daños, pero son insuficientes para eliminar el riesgo de infecciones derivadas de procesos y condiciones laborales que implican contacto directo con fluidos biológicos contaminados. Esta realidad valida la persistencia de las ausencias laborales incluso frente a los protocolos preventivos vigentes.

La NR-15^(1)^ vincula el reconocimiento de la insalubridad biológica en su grado máximo a la existencia de aislamiento físico del ambiente de trabajo, es decir, a un criterio de naturaleza arquitectónica que desconsidera la dinámica epidemiológica real de los servicios de salud, como las unidades de triaje, los servicios de urgencias y las unidades de emergencia, que son, por definición, espacios de contacto continuo e imprevisible con agentes patógenos^(6)^.

La exposición no está confinada a salas aisladas; es ubicua, difusa y constante durante toda la jornada laboral, ya que el profesional de enfermería actúa bajo un nexo causal permanente con agentes biológicos, donde la exposición constituye la norma y no una excepción geográfica. Por lo tanto, el criterio de “aislamiento” de la NR-15 fue concebido en un contexto hospitalario que no refleja la complejidad epidemiológica contemporánea^(6)^.

La premisa de que el riesgo infectocontagioso está restringido a las áreas de aislamiento es científicamente obsoleta. Estudios recientes^(3-4)^ demuestran que las unidades de triaje y los servicios de urgencias presentan cargas patógenas equivalentes o incluso superiores a las de las áreas de aislamiento, debido al flujo constante de pacientes sin diagnóstico previo y a la coexistencia de múltiples patógenos.

Manzi^(6)^, magistrado del Tribunal Regional del Trabajo, denuncia la invisibilidad del riesgo en los sectores de triaje hospitalario, donde la ausencia de un diagnóstico previo no elimina la peligrosidad biológica, sino que la hace más insidiosa. Afirma que la ciencia del cuidado exige que la insalubridad sea evaluada según la carga biológica y la naturaleza de la exposición, y no según delimitaciones arquitectónicas.

Complementariamente, Ezaias, Cardoso y Marziale^(7)^ fundamentan la necesidad de otorgar el grado máximo del adicional por insalubridad a los trabajadores de enfermería bajo el principio de la dignidad de la persona humana, argumentando que la protección de la salud del trabajador debe ser integral y que la diferenciación de los adicionales basada en criterios arquitectónicos vulnera la ética y la justicia distributiva. Según estos autores, la reflexión bioética sostiene que el adicional por insalubridad, bajo el principio de la dignidad de la persona humana, no debe considerarse como un precio pagado por la exposición al peligro, sino como un mecanismo que revela las deficiencias de las medidas de protección colectiva. La dignidad humana constituye el núcleo axiológico que debe orientar las NRs, impidiendo que la salud del profesional sea sometida a una lógica mercantilista en la que la vida sea valorada mediante porcentajes sobre el salario mínimo.

Por lo tanto, la reclasificación de la insalubridad constituye un imperativo de justicia social y seguridad sanitaria. La protección del trabajador de enfermería es inseparable de la calidad de la atención sanitaria prestada a la sociedad. Ha llegado el momento de alinear la legislación con la ciencia y transformar la evidencia en una agenda política que garantice dignidad y reconocimiento a quienes sostienen la salud pública brasileña.

La convergencia de evidencias científicas, fundamentos constitucionales, jurisprudencia favorable y movilización institucional conforma un argumento sólido y multidimensional, haciendo que la reclasificación de la insalubridad no solo sea legítima, sino también jurídicamente sostenible y políticamente inaplazable. Esta reclasificación representa no solo una conquista financiera, sino también el reconocimiento formal del riesgo real enfrentado diariamente por los profesionales de enfermería, constituyendo un paso estructural hacia la valorización de la categoría profesional.

El momento de la revisión técnica del Anexo 14 de la NR-15 ante el Ministerio de Trabajo es ahora. La inacción de la categoría representa la perpetuación de una injusticia normativa con impacto directo sobre millones de profesionales de enfermería. Lideremos juntos la producción de indicadores epidemiológicos que sustenten la revisión técnica del Anexo 14 de la NR-15 ante el Ministerio de Trabajo, transformando los datos en acción institucional y en una herramienta de apoyo para la movilización colectiva.
